# Road Feature Detection for Advance Driver Assistance System Using Deep Learning

**DOI:** 10.3390/s23094466

**Published:** 2023-05-04

**Authors:** Hamza Nadeem, Kashif Javed, Zain Nadeem, Muhammad Jawad Khan, Saddaf Rubab, Dong Keon Yon, Rizwan Ali Naqvi

**Affiliations:** 1 Engineering and Management Sciences, Balochistan University of Information Technology Engineering & Management Sciences, Quetta 87300, Pakistan; hamza.nadeem@buitms.edu.pk (H.N.); zain.nadeem@buitms.edu.pk (Z.N.); 2 School of Mechanical and Manufacturing Engineering, National University of Science and Technology, Islamabad 44000, Pakistan; kjaved@smme.nust.edu.pk (K.J.); jawad.khan@smme.nust.edu.pk (M.J.K.); 3 Department of Computer Engineering, College of Computing and Informatics, University of Sharjah, Sharjah 27272, United Arab Emirates; srubab@sharjah.ac.ae; 4 Center for Digital Health, Medical Science Research Institute, Kyung Hee University Medical Center, Kyung Hee University College of Medicine, Seoul 02447, Republic of Korea; 5 Department of Unmanned Vehicle Engineering, Sejong University, Seoul 05006, Republic of Korea

**Keywords:** deep learning, object detection, YOLOv7, Faster-RCNNs, computer vision, Driver Assistance, traffic signs

## Abstract

Hundreds of people are injured or killed in road accidents. These accidents are caused by several intrinsic and extrinsic factors, including the attentiveness of the driver towards the road and its associated features. These features include approaching vehicles, pedestrians, and static fixtures, such as road lanes and traffic signs. If a driver is made aware of these features in a timely manner, a huge chunk of these accidents can be avoided. This study proposes a computer vision-based solution for detecting and recognizing traffic types and signs to help drivers pave the door for self-driving cars. A real-world roadside dataset was collected under varying lighting and road conditions, and individual frames were annotated. Two deep learning models, YOLOv7 and Faster RCNN, were trained on this custom-collected dataset to detect the aforementioned road features. The models produced mean Average Precision (*mAP*) scores of 87.20% and 75.64%, respectively, along with class accuracies of over 98.80%; all of these were state-of-the-art. The proposed model provides an excellent benchmark to build on to help improve traffic situations and enable future technological advances, such as Advance Driver Assistance System (ADAS) and self-driving cars.

## 1. Introduction

The world has moved towards Industry 4.0—Artificial Intelligence; although, a few countries are still trying to catch up with the rest of the world. According to an article published by The News International on 21 November 2021, the past decade saw 104,105 road accidents that caused 55,141 deaths and 126,144 injuries [1]. A total of 120,501 vehicles were involved in these accidents, resulting in significant material and human life losses. The different causes of these accidents include not abiding by traffic rules, overspeeding, driver negligence, and blind spots. Therefore, measures must be taken to minimize road accidents by making cars smarter and enabling drivers to make better decisions by providing more information about roadside features. Another approach is to allow the smooth entry of self-driving cars into the local market and enable the technologies required for their efficient operation.

Self-driving cars have attracted the attention of scientists and the general public since the invention of automobiles. This trend escalated when cars became commercially available in the 1920 s; however, self-driving cars in their true essence came into being after Carnegie Mellon University started the Navigation Lab (NAVLAB) project in 1986 [2]. These cars are not easy to operate, especially in environments as chaotic as humans live in; hence, specialized sensing is required for their successful operation. These can be as basic as Radio Detection and Ranging (RADAR) and Global Positioning Systems (GPS) to more complex LiDARs (Light Detection and Ranging) and computer vision-based solutions, which sense the surroundings of people, cars, buildings, road lanes, and traffic signs.

Traffic-type detection is an important aspect of self-driving cars and assisted driving systems. Considering these applications, traffic must be detected and identified up to a certain degree of accuracy to ensure that vehicles and passengers are safe. Often, the type of traffic is not given sufficient value when considering driver and car safety. However, identifying the types of traffic is equally, if not more, important than simply detecting them to better assess any situation, assist in avoiding road accidents, and minimize the damage caused by them. Traffic signs are a universal means of enforcing traffic regulations, and self-driving cars must recognize them for safe operation. Furthermore, traffic signs differ worldwide. Therefore, indigenous datasets are required. There is a need to gather a diverse set of images from across the country under different lighting conditions and using various cameras and imaging modes. Appropriate labeling of the obtained data to enable accurate detection and classification of traffic signs and their respective types will establish a high-quality benchmark for subsequent research in the field.

The objective of this research is to address the practical challenges encountered in transportation systems and road infrastructure. These include, but are not limited to, the real-time monitoring of traffic around a driver’s vehicle using an Advanced Driver Assistance System (ADAS), which can later be evolved and geared towards self-driving cars and smart city initiatives. We propose a deep learning-based model for the detection and recognition of traffic signs and types. This model was trained on a dataset collected in the form of videos captured using different cameras and under different lighting conditions. Video keyframes were extracted from the collected videos and annotated using predefined class labels. The images (keyframes) were compiled into a single dataset. The model is composed of two modules: YOLOv7 for traffic type identification and RCNNs for traffic sign recognition. The model was then cross-validated and regularized to improve its performance. The final model was tested in real-world scenarios and thereafter tweaked according to the requirements.

The paper is further divided into the following sections: First, the current literature on the topic is reviewed in detail in the literature review section to extract the shortcomings and research gaps in relevant state-of-the-art solutions. Subsequently, based on an in-depth analysis of these issues, the process used to reach the solution is described along with the simulation setup in the methodology section, which also explains the process of data collection, data annotation, and all the preprocessing which has gone in to make the data for the detection models. The results section begins with a brief description of the performance metrics used to evaluate the performance of the model. The output of the models and the training, validation, and testing results of the performance metrics are also discussed. The discussion section revolves around novelty, improvements over the state-of-the-art methods, and possible future work in the research area. Finally, the conclusion section discusses the key achievements and outcomes of this research.

## 2. Literature Review

The concept of a system that assists drivers in possible blind spots and missed cues has been discussed as an easier way to automate and achieve autonomy. Some of the basic elements of such Driver Assistance Systems included sensing the environment for different obstacles, traffic signs, pedestrians, and other traffic fields [3,4,5,6]. Traditional image processing techniques have been used previously [7,8,9,10]. However, these methods are significantly slower than current state-of-the-art practices, which is a hindrance. Considering the application of our project, the accuracy and precision of the ADAS are paramount to the safety of users, vehicles, and road traffic. This issue has been addressed using a variety of techniques, such as vectorized IoU, as discussed in [11]. The issue of illumination has also been well researched, and multimodal fusions were utilized in [12], where infrared and visible region images were combined to improve feature extraction. Algorithms with evolutionary algorithm-based hyperparameter tuning have also been used to improve model performance [13]. Several conventional image processing-based approaches [14,15] have been used to identify traffic signs and types. The detection of traffic signs and types is a very time-sensitive process, where small delays can result in fatalities, and conventional techniques are not applicable here. This is because the slower processing times of these traditional techniques can become a bottleneck. These systems work based on visual features such as colors and shapes using algorithms such as color segmentation [16,17]. Other notable algorithms include scale-invariant feature transforms (SIFT), accelerated robust features (SURF), and binary robust invariant scalable keypoints (BRISK), among others [18,19,20]. In several recent studies, up to a certain degree of accuracy, convolutional neural networks (CNNs) have been deployed heavily in traffic detection and identification [21]. To avoid potential accidents involving traffic, such as forward collisions and vehicle overtaking, studies have been conducted to assist with safe lane-change operations using symmetry verification to detect lanes [22,23,24]. Research has also been conducted to prioritize certain traffic types [25]. This helps reduce the damage caused by road accidents, or in the cases of [26,27], helps ambulances and firetrucks avoid traffic jams with the help of smart city surveillance systems. Research on automated detection of traffic signs was initiated in Japan in 1984 [28]. Several other techniques that utilize the spatial aspects of an image, such as shape and color, have been employed in related research, and several publications support this idea [29,30,31,32].

More recently, learning-based algorithms have replaced them and have been successfully implemented in traffic type and traffic sign recognition problems, such as the use of CNNs of the German dataset (GTSRB) [33]. Convolutional neural networks (CNNs) require a large number of images to work efficiently, and many countries around the world lack a well-maintained dataset of traffic sign images. When working around learning-based techniques, a large number of images are required, and several organized datasets exist, such as the LISA traffic sign dataset comprising American traffic signs [34], the German traffic sign dataset [33], and the Belgian traffic sign dataset [35]. All these datasets have differing numbers of classes, but the common theme is the presence of a large number of images, such as the 39,000 images in the German Dataset mentioned above. Furthermore, these datasets are purposefully built, curated, and regularly used to inspire new learning-based models by pitting them against each other during competitions [28]. This has proved fruitful, as these competitions have resulted in the development of a great deal of the literature and state-of-the-art computer vision models, all of which seem to agree with the assumption that more data indicate better performance. A survey paper [36] published in 2017 summarized multiple approaches for traffic sign recognition used during the “Video and Image Processing Cup 2017”. These methods include traditional image processing as well as state-of-the-art deep learning, and convolution-based networks. The main theme of nearly all approaches discussed here is the need for several thousand images to work. Furthermore, these models work on a single-image basis and not on continuous video frames. Another study [37] introduced transfer learning using Inception-v3 and data augmentation on the Belgian traffic sign dataset to improve the accuracy to 99%. This was the start of the transfer learning trend in computer vision and directed the focus of research towards adopting deeper pre-trained networks and fine-tuning them on relatively smaller datasets. The implemented model already had thousands of images which were augmented to increase them further. In the case of some traffic sign datasets, the low number of images presents a bottleneck that even augmentation cannot be circumvent. A recent paper [38] presented an interesting comparison study. This study compares three different CNN architectures with a custom eight-layer CNN producing the best accuracy of 96%, while the other architectures used were VGG-16 and ResNet-50.

As is evident from the majority of research on traffic sign detection, the use of German/Belgian/US or similar well-maintained datasets with thousands of images is integral to producing good results. The same results were not replicable for all datasets because of the lack of available organized datasets. The basis of this research, specifically in terms of traffic signs in Pakistan, was established in 2014 by researchers from the National University of Computer and Emerging Sciences (NUCES), Islamabad, in their paper [6]. They collected a custom dataset and used color-based segmentation, followed by the application of the Hough transform to find circles, triangles, or rectangles in the images. Recognition was performed using the feature-matching techniques of conventional image processing, such as SIFT, SURF, and BRISK. This resulted in slow processing times owing to the long pipeline of implementation, and the model was not sufficiently robust, as accuracy decreased when the images were not cropped or contained a background. The second available work on Pakistani traffic sign images was published in 2018 [39], in which traffic signs were detected and identified through marker-based techniques. To introduce a holistic system, the recognition process is augmented with audio instructions for the driver. Although no detection technique or accuracy numbers are explicitly mentioned in this paper, a dataset was collected, and the system required significant improvements owing to the slow processing times of the proposed techniques. The first attempt to introduce learning-based algorithms in Pakistan was made in 2018 [40]. A dataset of 359 images was collected, cropped, and labeled according to the classes defined per the local standard. A model pre-trained on the German dataset was fine-tuned on the Pakistani dataset to induce transfer learning. The pilot project achieved an extremely low class accuracy of 41% owing to the small number of images (359). Authors from NUCES, Islamabad increased the number of images to 579, improving the class accuracy to 72%; however, significant overfitting was observed in this case [41]. These results were further improved through restricted overfitting [39].

The ADAS is anticipated to eliminate human error while driving to reduce traffic-related accidents [42]. These accidents are caused by several factors that must be considered, such as driving conditions and driver emotions [43]. However, observing driving conditions and classifying drivers’ behavior in real time poses several challenges: dizziness due to long drives, driver aggressiveness, and generally distracted drivers [44]. Deep learning-based ADAS can efficiently address these challenges by incorporating both remote sensing applications and the Internet of Things (IoT) [45]. Identification of traffic types is an important aspect of self-driving cars and assisted driving systems field [43]. Considering these applications, traffic must be detected and identified up to a certain degree of accuracy to ensure that vehicles and passengers are safe. Research on traffic detection and recognition has been conducted using different methods, including traditional image processing-based and learning-based algorithms.

A paper [25] published in 2020 proposed an improved version of the single-shot detector, MobileNet. Nearly all MobileNet-based architectures use ‘depthwise separable convolutions’, which employ two operations: depthwise and pointwise convolutions. A mean Average Precision (*mAP*) value of 91.20% was achieved; however, the model was only trained and tested for the detection of construction vehicles. Another [27] introduced an algorithm for vehicle detection based on YOLOv3 integrated with intelligent traffic lights. The use of YOLOv3 as the base model provided a lightweight design with low execution time. The proposed network was also used for traffic control after training in the Brazilian traffic code. However, this model works on a single-image basis and not on continuous video frames. A recent study [26] presented a different and interesting approach to traffic classification. A deep belief network (DBN)-based model was used to classify the vehicles into four categories: pedestrians, bikes, motorcycles, and vehicles. The proposed model achieved a high accuracy classification rate of 89.53%, considering that the model was trained using only 1000 images. However, this also raises a red flag because a small dataset can cause the model to have a sampling bias and underperform on outliers.

## 3. Methodology

### 3.1. Simulation Setup

The training process was performed using an NVIDIA Tesla P100 GPU provided by Google Colaboratory. The training was performed for 10,000 epochs/iterations to obtain precise and stable results. The training time was 2:35:11 (0.9313 s/s) for the GPU. Table 1 lists the technical specifications of the environment used for training and testing.

### 3.2. Data Collection

Deep learning-based algorithms require massive amounts of data for generalization. This is because of the inherent properties of modeling the available training data. This is an area in which several countries are lacking, and there is a severe shortage of available data. As part of the research, roadside videos were collected from across Pakistan to provide the algorithm with sufficient training data. Several datasets of traffic sign images are available worldwide and have been discussed in detail in [46]; however, there are no datasets available that contain sufficient frames relating to both traffic type and traffic signs.

**Figure 1:**
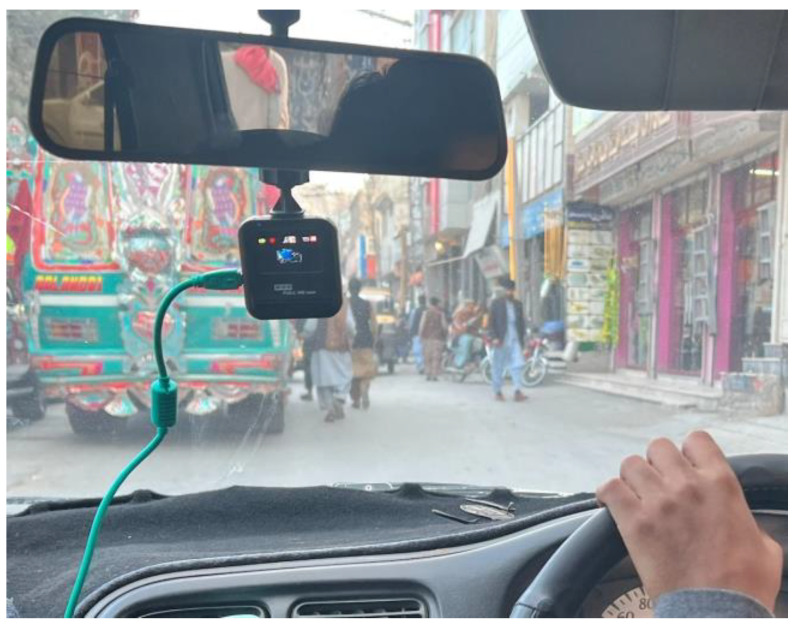
Experimental setup.

The data were collected in the form of videos, which were later converted into images by extracting video frames. The video recording device was mounted on the car windshield at a height of 1.20 m (off the ground) and the horizontal center of the car windshield, as shown in Figure 1. Different cameras have been used for videography, including smartphone cameras and dashcams. The smartphones used are iPhone 12, iPhone 13, Samsung Galaxy Note 10, and Samsung Galaxy S10, and the dashcam used is ‘HD High-Definition Vehicle Blackbox DVR’ with a maximum video resolution of full HD 1920 × 1080 and maximum image resolution of 12 megapixels.

A total of 30 videos were deliberately collected with varying properties, such as frame rate (fps), brightness, exposure, and lighting settings. These properties affect the results of the learning-based models. The frame rate is responsible for the number of frames extracted from each second and affects the total number of images. The total runtime of these 30 videos amounted to 5 h, 42 min, and 1 s. Of these 30 videos, 23 were collected from a few cities in Pakistan, including but not limited to Quetta, Karachi, Lahore, Islamabad, and Rawalpindi, with 2 h, 35 min, and 58 s of video footage. Further videos were fetched from various open-source video-sharing platforms and had a total runtime of 3 h, 6 min, and 3 s.

### 3.3. Preprocessing

After data collection, the next step began with the extraction of individual frames from the video footage. Considering the various frames in each video and the total runtime, all videos equated to approximately 0.56 million frames/images. Although it was stated above that the number of training images is generally directly proportional to the model performance, in this specific case, many of the extracted frames had little or no visual change and would only require expensive processing. This is because the spatial features present in adjacent frames are typically similar and do not add any benefits.

To cater to this, ‘keyframes’ were extracted according to equation (1). This resulted in 109,463 final frames. Furthermore, the input dimensions of all images/frames passed on to any learning-based algorithm must remain constant. In this study, the resolution was fixed at 640 × 380 pixels. Another reason for setting the resolution to this specific value is that the videos differed in aspect ratios and resolutions, some of which were very high. A higher resolution, while providing better spatial features, also implies a longer training time and the use of precious computational resources. After a certain point, it becomes important to examine the cost–benefit analysis of the input resolution. It is generally observed that after a certain resolution, any increase will return a negligible increase in the model performance, but it will take a significantly longer time to train.

### 3.4. Data Annotation

After preprocessing the video to the set requirements, the frames were annotated for the presence of relevant objects. This is important because annotations are labels passed to the deep learning model in a supervised learning scenario. The annotations were performed using a Computer Vision Annotation Tool (CVAT) from Intel. It can output annotations in various formats depending on the type of model being trained. The types of objects to be detected were divided into five main classes: four for traffic types and one for traffic signs. The four types of traffic considered in this study were pedestrians, bikes (bicycles and motorbikes), Light Transport Vehicles (LTVs), and Heavy Transport Vehicles (HTVs). The traffic sign superclass was further divided into 35 subclasses based on the exact type of traffic sign, as listed in Table 2.

### 3.5. Flow Diagram

**Figure 2:**
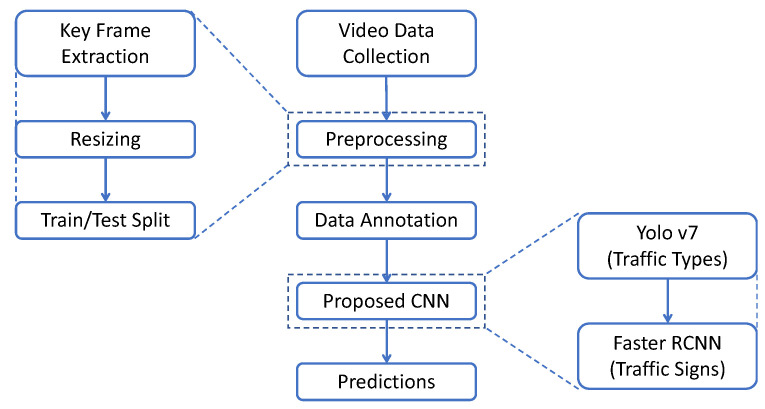
Flow diagram.

The overall flow of data and all individual steps are shown in the flow diagram in Figure 2. The process begins with key preprocessing, as detailed in Section 3.3, and includes keyframe extraction, resizing, and training/test split steps. The training/test split is performed to distribute the data into two parts: one used for training the data and the other used to test the performance of the model by emulating real-world conditions where the model encounters unseen traffic signs and types. All frames were randomly sorted at a ratio of 80:20 for the training test. The preprocessed data were annotated and passed to the proposed CNN.

The proposed CNN performs three steps in general: using varying techniques based on the type of CNN being used, extracting relevant features, detecting objects, and classifying them. Two CNNs were used for the two distinct tasks of the research: Yolo v7 for traffic type and Faster-RCNN for traffic sign recognition, which are further detailed in the next section. Consequently, predictions are made and further decisions are made based on them.

### 3.6. Model Architecture

In this study, the two models were used for object detection in two distinct tasks. One of the models used was Faster-RCNN, which was used to detect traffic signs and their subclasses. The other model was YOLOv7, which was used to detect traffic types. Both models are detailed below.

#### 3.6.1. Faster-RCNN

The RCNN—region-based convolutional neural networks (CNNs)—are a family of CNNs based on the idea of dividing an image into regions and then detecting objects in them, instead of the whole image. There are multiple types and flavors of RCNNs; however, the inherent concept remains the same and consists of three main modules. The first module in the vanilla RCNN proposed 2000 regions from the input image using a selective search algorithm. After the regions were extracted and resized, the preceding module extracted features from each region in the form of a vector of size 4096 × 1. This feature vector is then passed to the classifier layer, which, in this case, is a Support Vector Machine (SVM) pre-trained on a publicly available image dataset. The SVM then classifies the region into one of the predefined object classes or as background if it does not detect any object in the given region.

The Faster-RCNN which is used in this study differs considerably from the vanilla RCNN in that it changes the architecture into a two-module implementation. The first is the Region Proposal Network (RPN) and the second is the classifier network. The performance of the model was improved because the most computationally expensive task of calculating the convolution feature maps was used by both networks. Faster RCNN uses a RPN instead of a selective search algorithm, which is a sizeable improvement over the traditional neural network that is now used to generate proposals with various sizes and aspect ratios. Since the feature maps remain constant throughout the network pipeline, the RPN deduces an “objectness” score for each region. This indicates the probability of an object being present in a region. The Region of Interest (ROI) pooling layer uses previously extracted feature maps to pool the proposed regions, and classification was performed only on regions with a high probability of having an object. This further improves the detection speed because only a fragment of all the regions is passed on to the classification layer.

**Figure 3:**
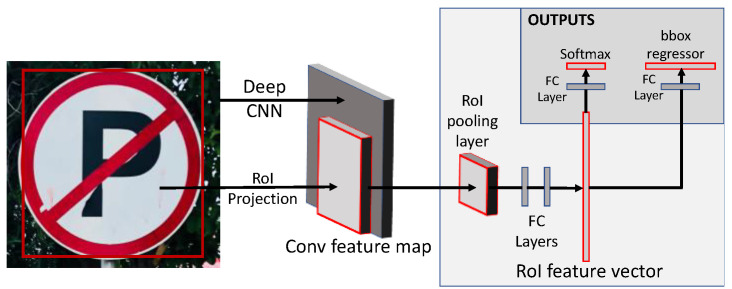
Model architecture of Faster-RCNN [47].

Faster RCNNs use a backbone CNN that can be modified depending on the application and requirements. The most commonly used backbone networks are ResNet-50/101, VGG-16/19, RetinaNet, and Xception. Figure 3 depicts the model architecture.

#### 3.6.2. YOLOv7

**Figure 4:**
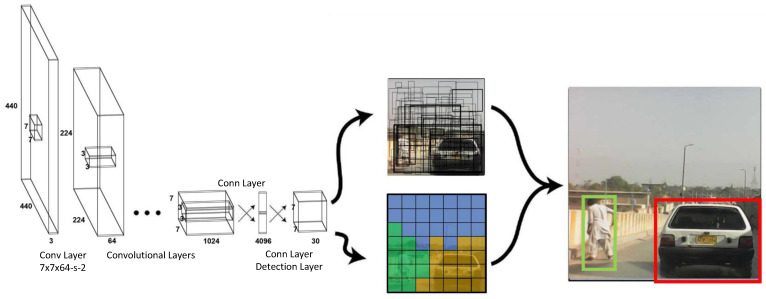
Model Architecture for YOLOv7 [48].

The first version of the YOLO object detector was introduced in 2015 in a paper titled “You Only Look Once: Unified, Real-Time Object Detection”—YOLOv1 [40]. Since then, multiple versions and flavors of the base model have been released. The seventh version was presented in the paper titled “YOLOv7: Trainable bag-of-freebies sets new state-of-the-art for real-time object” [48]. In this study, the YOLOv7 architecture shown in Figure 4 was used for the detection and identification of traffic types. This makes the predictions for the bounding boxes more accurate than those of their predecessors at similar inference speeds.

YOLO is a regression-based algorithm, but instead of selecting important features of an image, it predicts bounding boxes and labels/classes for the image. The most significant element of this algorithm is that it performs all this in a single run, as suggested by “You Only Look Once.” Ultimately, the aim is to predict the object class and specify the location of the object using a bounding box.

The YOLO-based model, on which every version is based, comprises three main modules:

- Residual block: The first module considers the input image and divides it into various grid cells; typically, the size is 19 × 19. Each grid cell is responsible for detecting objects that may appear within it based on the location of the center of the object.
- Bounding Box Regression: The second module uses single bounding box regression on any detected object. This provides the probability that an object will appear in the bounding box, that is, the outline or boundary that highlights the location of the object in the input image. For each bounding box, there are four attributes to predict: center (x, y), width, height, and class.
- Intersection over Union (IoU): The third module, using the concept of IoU, describes how the bounding boxes overlap and provides an output box in which the objects are perfectly surrounded. Every grid cell is tasked with predicting the bounding boxes and their probability or confidence score. If the prediction for the bounding box is the same as that for the real bounding box, the IoU value equals one; consequently, any predicted bounding box that is not equal to the real bounding box is eliminated.

### 3.7. Advanced Driver Assistance System

An ADAS is designed as an active system to assist in a wide array of tasks while driving. Some of these tasks include but are not limited to

- Lane-keeping;
- Overtaking vehicles;
- Jaywalking pedestrians;
- Potential blind spots;
- Negligent/distracted drivers.

The ADAS Unit consists of two modules. The first module divided each video frame into a grid of 3 × 2 cells. For every predicted bounding box center (bx, by), their location is identified in the grid, along with the predicted labels for the traffic type. Using the labels and bounding box locations, the second module provides specified instructions for the detected conditions (e.g., traffic in front, overtaking vehicles). Table 3 shows the list of instructions provided as assistance to the driver by the ADAS according to the different situations detected in decreasing order of priority.

## 4. Results

### 4.1. Performance Metrics

It is pertinent to mention the performance metric being used to characterize the predictions of the model. The primary metrics are true positive (*TP*), false positive (*FP*), true negative (*TN*), and false negative (*FN*) values. These results are presented in Table 4.

Another aspect that must be considered when using an object detection model is the Intersection over Union (IoU). IoU is a measure of how much the predicted bounding box overlaps the original—or ground truth—bounding box in the case of a prediction being made, i.e., it is a ratio of the “area of overlap” and the “total area covered by the original and predicted bounding boxes” combined, as given by Equation (1).

IoU = Intersection of bounding box areas/union of bounding box areas (1)

This metric was used along with a threshold to classify bounding using one of the primary metrics. Therefore, if a bounding box is below the required threshold, it is classified as a false positive because it makes a prediction, but that prediction does not have sufficient quality to be called a correct prediction or true positive. The IoU threshold can vary depending on various situations and applications, as well as the size of the object under observation, but the default or generally accepted value is set at 0.5. Furthermore, the primary metrics are combined to form the secondary metrics of precision and recall, as given by Equations (2) and (3).

(2) $$Precision=\frac{TP}{TP+FP}$$

(3) $$Recall=\frac{TP}{TP+FN}$$

Precision is a measure of the accuracy of a model’s predictions, that is, the number or percentage of correct predictions made by the model relative to the total number of predictions made. The recall is a measure of how well the model predicts the presence of objects, that is, the number or percentage of objects detected among all objects present.

This is then succeeded by a tertiary metric called the average precision, which is generally defined as the area under the precision–recall curve. This can be calculated by simple integration, as shown in Equation (4). The main metric used to characterize the findings of this study was mean Average Precision (*mAP*). The *mAP* is the cumulative mean of the average precision across all classes of the object being predicted and is given by Equation (5) [49].

(4) $$p_{interp}\left(r\right)=\underset{\tilde{r}\geq r}{\max}p\left(\tilde{r}\right)$$

(5) $$mAP=\frac{1}{n}\sum_{k=1}^{k=n}{AP}_{k}$$

where, ${AP}_{k}=the\;AP\;of\;class\;k$, n=the number of classes.

### 4.2. Numerical and Graphical Results

As mentioned above, this research is divided into two parts: the detection of traffic types and the detection and classification of traffic signs. The results for these individual parts are presented below.

#### 4.2.1. Traffic Type Recognition

Class accuracy and loss and various *AP* scores are shown, including mAP@0.5, which is the mean Average Precision at 50%. IoU and mAP@0.5:0.95 averages the results over multiple IoU values and combines them into a single metric to provide a consolidated value. Table 5 and Table 6 present a comparison between the two YOLO models and the performance of YOLOv7 on traffic-type detection tasks, respectively.

The graphical results of several other common and useful metrics for the top-performing model (YOLOv7) are presented below.

**Figure 5:**
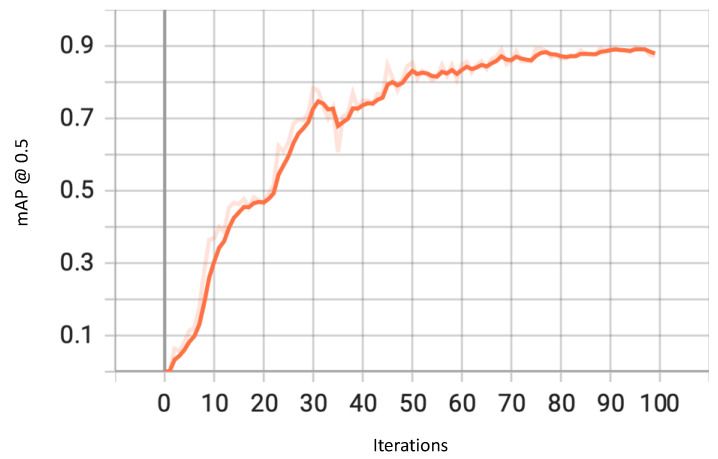
mAP@0.5 for YOLOv7.

The *mAP* is the cumulative mean of the average precision across all classes of objects being predicted. As shown in the graph in Figure 5, the *mAP* over the IoU threshold of 0.5 improves consistently and ultimately flattens out after approximately 70 iterations, with a final value of 87.20%.

**Figure 6:**
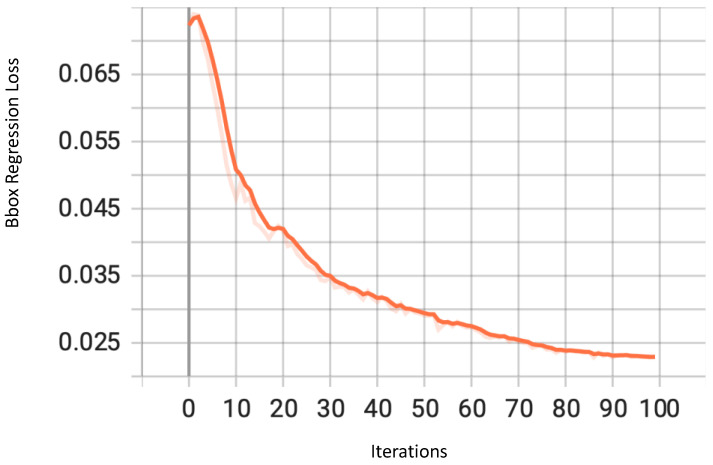
Bounding box regression loss for YOLOv7.

As mentioned in Section 3.6.2, the bounding box coordinates were calculated through regression in a branch of the network using the mean square error. The loss of this regression is a strong indicator of how well these boxes are ‘bounding’ the objects. The values of the regression loss decreased continuously to a very low value, showing improvements as the training progressed, as shown in Figure 6.

**Figure 7:**
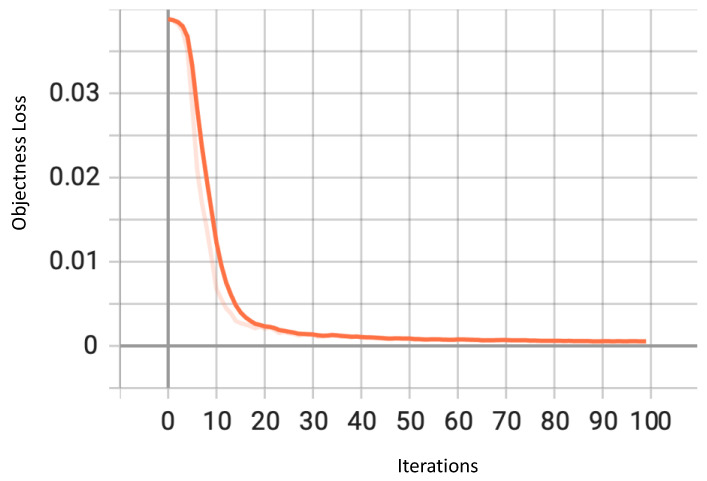
Objectness loss for YOLOv7.

Objectness loss is another major identifier of model performance. Objectness is a relatively new term in the field of performance metrics. This can be described as the confidence that a network has an object existing in a predicted bounding box. Objectness Loss helps the network predict the correct IoU using binary cross-entropy. The graph in Figure 7 shows a steady decrease as the number of iterations increased, indicating an improvement in the model’s performance.

**Figure 8:**
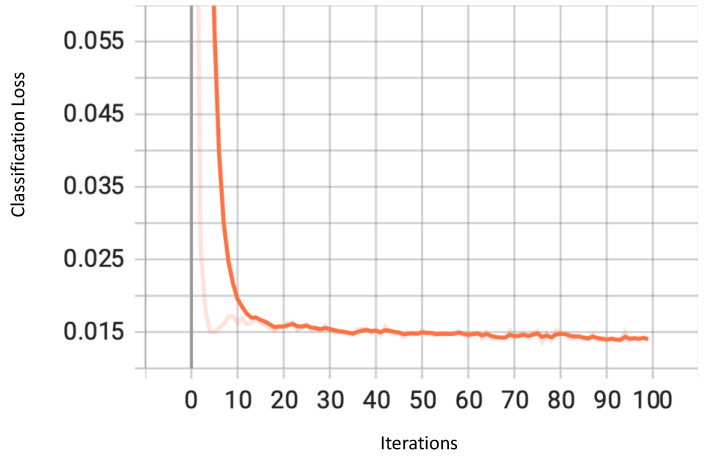
Classification Loss for YOLOv7.

YOLOv7 classifies the detected objects into predefined classes, and this part of the architecture uses cross-entropy loss. As is evident from Figure 8, the loss generally trends downwards quite early and remains steady after approximately 40 iterations.

#### 4.2.2. Traffic Sign Recognition

Traffic sign images were trained on multiple state-of-the-art networks and the model architecture of the Faster-RCNN family, along with several different backbone networks. All the selected networks showed remarkable results and groundbreaking *mAP* numbers on the COCO dataset over the year. As shown in Table 7, the Faster RCNN model architecture with ResNet-101 and the Feature Pyramid Network as the backbone network produced the highest values for all relevant benchmarks. Here, the “*AP* | 50” means that the IoU value is more than 50%, and “*AP* | s/m/l” means the average precision of small, medium, or large objects.

**Figure 9:**
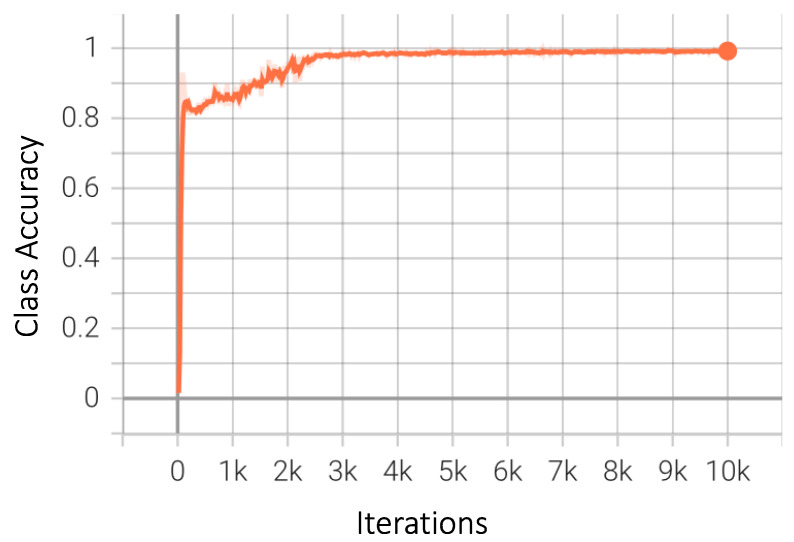
Average class accuracy for Faster-RCNN.

**Figure 10:**
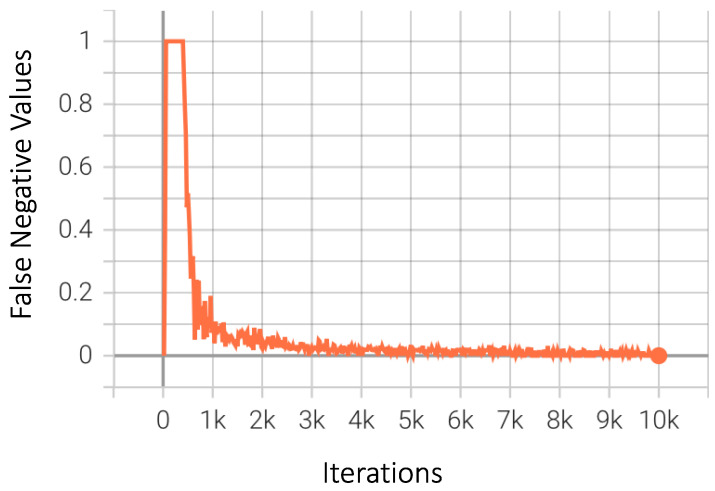
False negative values for Faster-RCNN.

Graphical results of several other common and useful metrics for the best-performing model are presented. As shown in Figure 9, the class accuracy flutters in the initial 1000 iterations, after which it starts to improve; it ultimately flattens after approximately 5000 iterations, with a final accuracy score of 98.57%. The false negative value is also an important metric, as previously explained in detail. The final value for false negative samples should be as low as possible for the model to function efficiently. This is exactly the case in our best-performing model, for which the graph in Figure 10 shows a steady decrease as the model extracts better features for detection.

**Figure 11:**
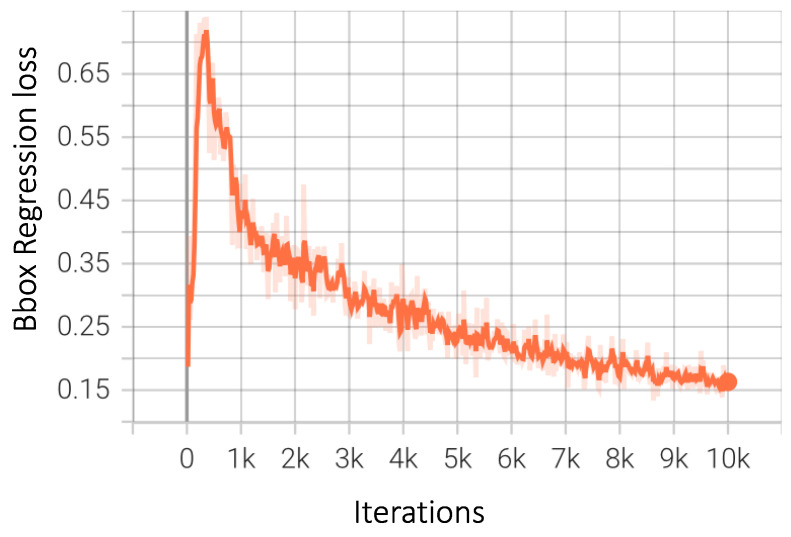
Bounding box regression loss for Faster-RCNN.

**Figure 12:**
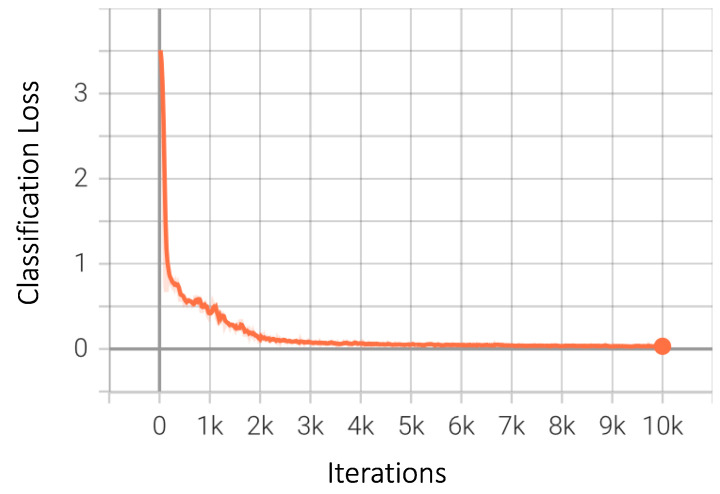
Classification loss for Faster-RCNN.

As previously discussed, bounding box regression loss is a strong indicator of how well these boxes are ‘bounding’ the objects. The values of the regression loss decreased continuously to a negligible value, showing improvements as the training progressed, as shown in Figure 11. In the Faster-RCNN family, the network is divided into two branches after the final fully connected layer. One of these branches is the classification branch, which classifies the detected objects into one of the predefined classes. This part of the architecture uses softmax, and the loss generally trends downwards quite early and remains steady after approximately 4000 iterations, as shown in Figure 12.

**Figure 13:**
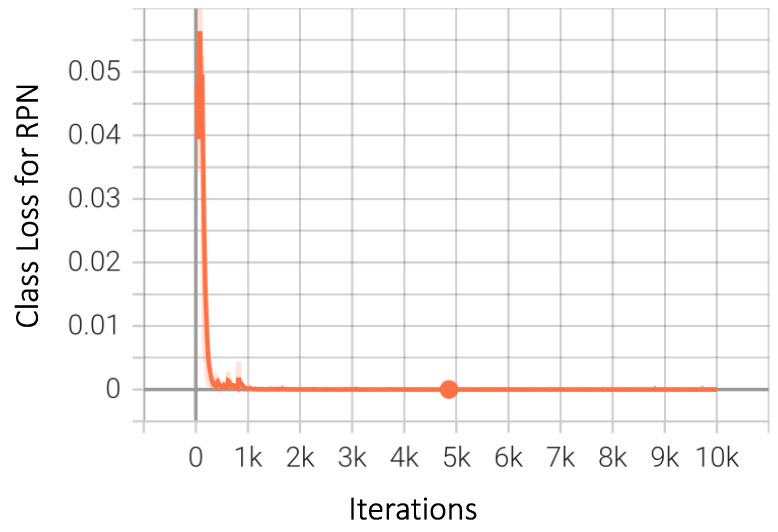
Class loss of Region Proposal Network (RPN) for Faster-RCNN.

**Figure 14:**
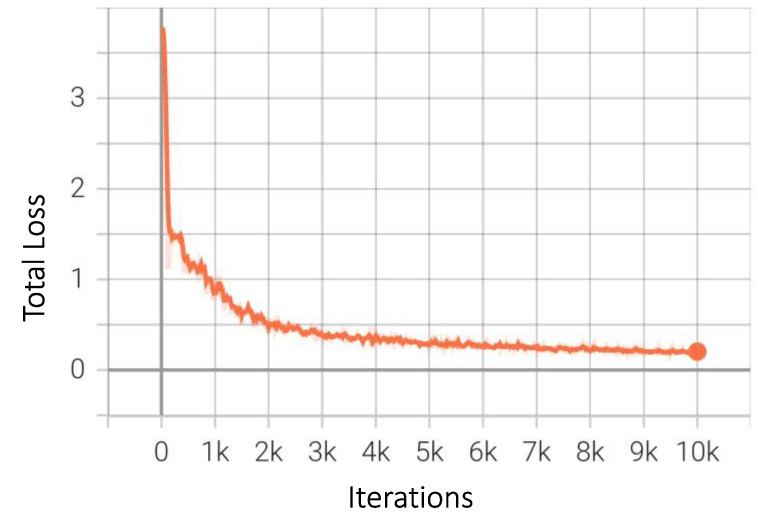
Total loss for Faster-RCNN.

As explained in the model architecture subsection above, Region Proposal Network is an integral part of the working of Faster-RCNN that we have used in this study. It is a standalone network, and Figure 13 shows the expected downwards trend of the network loss as the number of iterations increases. Model loss or error is another major identifier of model performance; in simple terms, it is the cumulative sum of the differences between the actual and predicted values. The graph in Figure 14 shows a steady decrease as the number of iterations increased, indicating an improvement in the model’s performance. This is the total loss, which includes both the bounding box regression loss and classification loss discussed above.

## 5. Discussion

The novelty of this study lies in the collection of vast amounts of new data regarding traffic signs and their types. This data have been collected from several cities while having a diverse range of visual features and artifacts. These include images with varying exposure ranges, brightness levels, and occlusions. Furthermore, this dataset is annotated for traffic type and sign recognition and can be used by researchers to develop and improve road models.

Furthermore, this is a pioneering study on the implementation of multiple object-detection-based systems to recognize traffic types and signs in countries without compiled datasets. This study is the first of its kind to have a model trained on frames from video footage of the streets. It also scored impressively on all performance metrics used internationally to characterize related models. This study was intended as an initial method for enabling better traffic conditions. Although the research has resulted in favorable outcomes and performance metrics, there is always room for improvement. The performance metrics can be improved using data augmentation to account for class imbalance. More training iterations/epochs can be used in case of the availability of better computational resources. Another case of improvement could be the use of all the frames instead of extracting keyframes and using them at higher resolutions to improve the results.

Object detection is a popular research topic, and recently published models can be used to obtain higher performance scores. Masked RCNN and image segmentation models can also be used to obtain pixel-wise detection but would require an even more tedious annotation process and could result in better real-world results when deploying the model. This research could be compounded by the development of specialized hardware to help turn the research project into a commercially viable product.

## 6. Conclusions

In this study, we present our Advanced Driver Assistance System that uses predictions from our detection and identification models, trained and tested on novel collected datasets. During this study, a first-of-its-kind dataset was collected from the roads of Pakistan and across various cities, including, but not limited to, Islamabad, Quetta, Lahore, and Karachi. The dataset comprised 5 h, 42 min, and 1 s of video footage and 109,463 keyframe images. The footage was annotated using rectangular bounding boxes and five distinct classes: pedestrians, bikes, LTVs, HTVs, and traffic signs. The traffic-sign class was further divided into 35 subclasses. Consequently, two models were trained: one for traffic types and the other for traffic signs. A YOLOv7 model was used to detect and identify traffic types and a faster RCNN model was used to detect and classify traffic signs. The *mAP* of YOLOv7 was up to 87.20% for all classes and the overall class accuracy was up to 98.8% with a *mAP* of over 75.63%.

## Figures and Tables

**Table 1:** Simulation setup detail.

Aspect	Detail
Operating system	Ubuntu 18.04.5 LTS
GPU	Tesla P100—PCIe—16 GB
RAM	26 GB
Programming language	Python 3.7.10
CUDA	Version 10.2.228
PyTorch	1.10.0

**Table 2:** List of traffic signs in Dallas.

S. No.	Traffic-Sign Name
1	Bridge Ahead
2	Crossroads
3	Give Way
4	Left Bend
5	No Horns
6	No Left Turn
7	No Mobile Allowed
8	No Overtaking
9	No Parking
10	No right turn
11	No U-Turn
12	Parking
13	Pedestrians
14	Railway Crossing
15	Right Bend
16	Road Divides
17	Roundabout Ahead
18	Sharp Right Turn
19	Slow
20	Speed Breaker Ahead
21	Speed Limit (20 kmph)
22	Speed Limit (25 kmph)
23	Speed Limit (30 kmph)
24	Speed Limit (40 kmph)
25	Speed Limit (45 kmph)
26	Speed Limit (50 kmph)
27	Speed Limit (60 kmph)
28	Speed Limit (65 kmph)
29	Speed Limit (70 kmph)
30	Speed Limit (80 kmph)
31	Steep Descent
32	Stop 1
33	Stop 2
34	U-Turn
35	Zigzag Road Ahead

**Table 3:** ADAS Instructions.

#	ADAS Instructions
1	No traffic *detected;* assistance not required
2	Pedestrian right in front of you, stop immediately
3	Bike right in front of you, slow down immediately
4	LTV right in front of you, slow down immediately
5	HTV right in front of you, slow down immediately
6	Pedestrian in front of you, slow down immediately
7	Bicycle in front of you, slow down slightly
8	LTV in front of you, stay cautious
9	HTV in front of you, slow down slightly
10	Pedestrian near your left side, watch out
11	Pedestrian near your right side, watch out
12	Bike overtaking from your left side, stay cautious
13	Bike overtaking from your right side, stay cautious
14	LTV overtaking from your left side, stay cautious
15	LTV overtaking from your right side, stay cautious
16	HTV overtaking from your left side, stay cautious
17	HTV overtaking from your right side, stay cautious
18	Pedestrian on your far-left side, watch out
19	Pedestrian on your far-right side, watch out
20	Bike on your far-left side, be careful while overtaking
21	Bike on your far-right side, be careful while overtaking
22	LTV on your far-left side, be careful while overtaking
23	LTV on your far-right side, be careful while overtaking
24	HTV on your far-left side, be careful while overtaking
25	HTV on your far-right side, be careful while overtaking

**Table 4:** Performance Metric.

Performance Metric	Description
True positive	It is when a model makes a prediction and correctly identifies the object
False positive	It is when a model makes a prediction even though no object was present
True negative	It is when a model does not make a prediction when there is no object
False negative	It is when a model does not make a prediction even though an object was present

**Table 5:** Performance metrics comparison for the trained models.

Architecture	P	R	mAP@0.5	mAP@0.5:0.95
YOLOv5	87.70%	62.31%	74.64%	43.01%
YOLOv7	87.61%	73.03%	87.21%	57.94%

**Table 6:** Performance metrics for YOLOv7.

YOLOv7				
Class	P	R	mAP@0.5	mAP@0.5:0.95
All	87.61%	73.03%	87.21%	57.94%
Pedestrian	92.53%	69.12%	84.11%	54.82%
Bike	93.02%	78.41%	89.94%	56.44%
HTV	81.83%	50.00%	83.41%	45.21%
LTV	95.90%	67.42%	87.32%	56.04%

**Table 7:** Performance metrics for Faster-RCNN model.

Model	*mAP*	*AP* | 50	*AP* | 75	*AP* | s	*AP* | m	*AP* | l
Faster-RCNN (R50-FPN)	70.27	98.36	84.60	53.83	72.58	84.55
ResNet-50 (Dilated Convolutions)	73.21	96.69	87.37	49.59	77.04	**85.51**
Xception-101	72.29	72.29	72.29	72.29	72.29	72.29
**Faster RCNN** **(R101-FPN) | train**	**75.64**	**99.09**	**92.62**	**65.67**	**77.64**	84.65
Faster-RCNN (R50-FPN) | val	49.70	64.61	59.15	46.95	40.18	69.61
Faster-RCNN (R50-FPN) | test	43.45	58.24	52.69	15.00	44.76	38.12

## Data Availability

Not applicable.
